# Psychometric validation of the Serbian version of the Fear Avoidance Component Scale (FACS)

**DOI:** 10.1371/journal.pone.0204311

**Published:** 2018-09-24

**Authors:** Aleksandar Knezevic, Randy Neblett, Robert J. Gatchel, Milica Jeremic-Knezevic, Vojislava Bugarski-Ignjatovic, Snezana Tomasevic-Todorovic, Ksenija Boskovic, Antonio I. Cuesta-Vargas

**Affiliations:** 1 Faculty of Medicine, University of Novi Sad, Novi Sad, Serbia; 2 Medical Rehabilitation Clinic, Clinical Centre of Vojvodina, Novi Sad, Serbia; 3 PRIDE Research Foundation, Dallas, Texas, United States of America; 4 Center of Excellence for the Study of Health & Chronic Illnesses, Department of Psychology, College of Science, The University of Texas at Arlington, Arlington, Texas, United States of America; 5 Department of Physiotherapy, Faculty of Health Science, University of Malaga, Malaga, Spain; 6 School of Clinical Science, Faculty of Health at the Queensland University of Technology, Brisbane, Australia; University of Indianapolis, UNITED STATES

## Abstract

**Objective:**

The Fear Avoidance Components Scale (FACS) is a new patient-reported outcome (PRO) questionnaire designed to comprehensively evaluate fear avoidance (FA) beliefs and attitudes in persons with painful medical conditions. The original English version has demonstrated acceptable psychometric properties, including concurrent and predictive validity. Two factors have been identified: 1. general fear avoidance; and 2. types of activities that are avoided.

**Methods:**

The FACS was first translated into Serbian, and then psychometrically validated. A cohort of 322 chronic musculoskeletal pain subjects completed the FACS-Serb and additional FA-related patient-reported outcome (PRO) measures. Their FACS-Serb scores were then compared to a cohort of 68 acute pain subjects.

**Results:**

Test-retest reliability (ICC2,1 = 0.928) and internal consistency for both Factors (Cronbach α 0.904 and 0,880 respectively) were very good. An acceptable fit was found with a confirmatory factor analysis of the 2-factor model found with the original English version of the FACS. Strong associations were found among FACS-Serb scores and other PRO measures of pain catastrophizing, depressive/anxiety symptoms, perceived disability, and pain intensity (p<0.001 for all analyses). FACS-Serb total scores, separate Factor scores, and subjective pain ratings were significantly higher in the chronic vs. acute pain cohorts (p<0.001 for all analyses).

**Conclusions:**

The FACS-Serb demonstrated strong psychometric properties, including strong reliability and internal consistency, criterion validity (through associations with other FA-related PRO measures), and discriminant validity (through comparisons with a separate acute pain cohort). The FACS-Serb appears to be a potentially useful pain-related assessment tool.

## Introduction

The fear-avoidance (FA) model has been proposed as one explanation of the transition from acute to chronic pain [1, 2]. This model was first introduced by Lethem et al. [2], and then further advanced by Vlaeyen et al. [1, 3]. More recent approaches to the FA model delineate additional complex factors that can influence whether recovery will prevail over disability in chronic pain patients [4]. In the basic FA model, if an acute pain is perceived as a threat, and one begins to catastrophize, then pain-related fear can evolve. This can then lead to avoidance of daily activities, hypervigilance of physical sensations, depressive symptom, physical disuse, deconditioning, and eventual disability [4, 5].

Because FA is associated with disability and negative treatment outcomes, identification and quantification of FA can be clinically useful [5]. A number of patient-reported outcome (PRO) questionnaires are available for assessing FA beliefs and attitudes [6–16], although four are the most often used: Tampa Scale for Kinesiophobia (TSK) [15]; Pain Catastrophizing Scale (PCS) [16]; Pain Anxiety Symptoms Scale (PASS) [7]; and Fear-Avoidance Beliefs Questionnaire (FABQ) [6]. Each of these instruments assesses some aspects of the current FA model, but all of them were developed before the model was fully developed, so none assesses all components of the model (i.e., cognitive, emotional, and behavioral). They have been criticized for psychometric weaknesses, including questionable construct validity, poor item-specificity, and lack of evidence-based cutoff values [17]. As the number of pain specialists accepting and realizing the importance of the FA concept increases, it appears increasingly important to have a PRO instrument that assesses all of the important components of the current FA model [1, 4]. Neblett et al. (2016) have developed a new instrument, the *Fear Avoidance Component Scale* (FACS), in an attempt to overcome the deficiencies of the previous FA-related PRO measures [18]. Acceptable reliability and internal consistency were found in its initial psychometric evaluation [18]. Five severity levels have been defined for clinical interpretation. The FACS has demonstrated that it can be a useful tool for predicting physical performance, other patient-reported symptoms of distress, and relevant post-treatment work outcomes in patients with chronic musculoskeletal pain [19].

Serbia is a relatively small country (about 7 million inhabitants, according to a 2011 Census of Population) [20]. However, as has been found in other countries, including the United States [21], a large percentage of the Serbia population report problems with pain. For instance, results of the 2013 National Health Survey of the Republic of Serbia found that 45% of the population reported physical pain in the 4-week period prior to the Survey, and 10% of population reported that pain had a strong effect on their usual daily activities [22]. The FACS appears to be a potentially useful new pain assessment tool for use in Serbian healthcare, but it is not currently available in the Serbian language. In fact, the only FA-related measure currently available in the Serbian language is the Pain Catastrophizing Scale. Hence, the goals of this study were to: translate the FACS into Serbian; evaluate its psychometric properties; and make the FACS-Serb available to Serbian-speaking healthcare personnel who wish to evaluate pain-related fear-avoidance.

## Materials and methods

This study consisted of two parts. First, the English version of the FACS was translated into Serbian, and then back-translated. Next, the Serbian version of the FACS was psychometrically-evaluated in a group of chronic pain patients and a control group of acute pain subjects. The study was approved by the Ethical Board of Clinical Centre of Vojvodina. All subjects signed an informed consent before they were enrolled in the study.

### Fear avoidance component scale (FACS)

The FACS is intended to comprehensively evaluate cognitive, behavioral, and affective components of FA in patients with painful medical conditions. Each item is scored on a 6-point Likert scale, from 0 “completely disagree” to 5 “completely agree.” Total scores, from 0 to 100, indicate one of the following severity levels: Subclinical (0–20); Mild (21–40); Moderate (41–60); Severe (61–80); and Extreme (81–100) [18].

### Translation process

The transcultural adaptation and validation process of the FACS was performed according to the recommendations of the International Society for Pharmacoeconomics and Outcomes Research (IPSOR) Guidelines [23]. Three native Serbian speakers, including the first author (AK) on this paper and two professional English-to-Serbian translators, performed an English-to-Serbian translation. Each translation was performed separately, and blinded from the other participants, producing three Serbian versions of the FACS (FACS-1, FACS-2, and FACS-3). After discussion, the three participants reconciled the translations into a single Serbian FACS. A backward Serbian-to-English translation was then performed by a professional Serbian-to-English translator and a native English speaker, who was unfamiliar with the original English version of the FACS and with the current research concept. A native English speaker, who was also one of the original developers of the FACS, and second author (RN) on this present study, evaluated the back translation against the original FACS for discrepancies. Based on this evaluation, a correction was made to one word one of the items. The Serbian translation was then pre-tested in a sample of eight patients with chronic pain syndrome to evaluate their comprehension of the items. These patients were recruited from the Medical Rehabilitation Clinic in the Clinical Centre of Vojvodina (Serbia), and included both genders, various education levels, and a range of ages. Based on the comments of these volunteers, which were assessed within a cognitive debriefing format, it was determined that no further changes were needed. This final version of the FACS-Serb can be found in S1 Appendix. A pdf of the FACS-Serb, and other translated versions of the FACS, can also be found at http://www.pridedallas.com/questionnaires.

### Subjects

**Fig 1:**
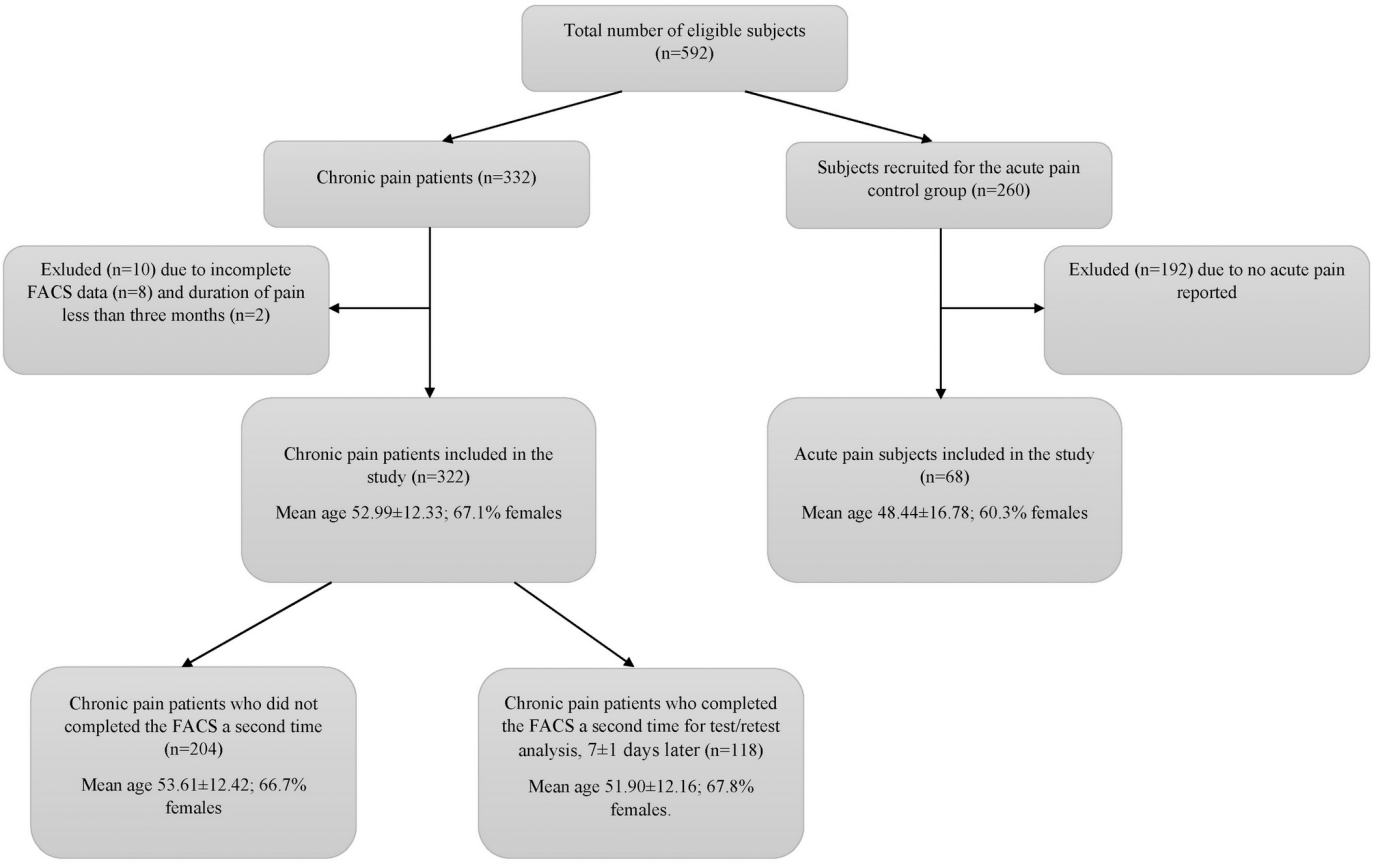
Flowchart of subjects.

This research included 332 chronic pain patients who were recruited from a medical rehabilitation program at the Medical Rehabilitation Clinic in the Clinical Centre of Vojvodina (Serbia), and who agreed to participate in the FACS data collection portion of the study. Chronic pain was defined as pain lasting for three or more months. Data collection was conducted from June 2016 until December 2016. All patients had been referred to the Clinic from both a primary healthcare institution in the City of Novi Sad, and from several other clinics in the Clinical Centre of Vojvodina (including neurosurgery, orthopedic surgery and traumatology, maxillofacial and oral surgery, and neurology). Exclusion criteria were: duration of pain less than 3 months; age < 18 or >75 years old; and subjects with poor Serbian language comprehension. All of the 332 patients completed a battery of PRO questionnaires, but 10 subjects were excluded from the final analysis. Eight were excluded due to incomplete FACS data, and two were excluded because the pain was not chronic (i.e. less than three-months). The remaining 322 patients were used for analyses. The study sample was 67.1% female, with an average age of 52.98±12.33, and a range of years-of-education (15.8% <9 years, 52.8% 9–12 years, and 31.4% >12 years). A variety of painful body areas and chronic pain disorders were represented, including low back pain (with sciatica included) (n = 124; 38.5%); cervical pain (including cervicobrachial syndrome) (n = 25; 7.8%); localized pain (shoulder, knee, hip, ankle, etc.) (n = 59; 18%); pain in two or more body parts (n = 64; 20%); complex regional pain syndrome (n = 5; 1.6%); and fibromyalgia (n = 30; 9.3%). All diagnoses were established by at least two physicians, including the referring physician and the treating physician at the Medical Rehabilitation Clinic. A subset of the total sample (n = 118), who were recruited between 1^st^ June and 1^st^ November, completed the FACS a second time, 7±1 days later, for the purpose of a test/re-retest analysis (see Fig 1). Patients in this period did not receive any new therapy.

To determine associations among FACS scores and other patient-reported clinical variables (convergent validity), patients were divided into FACS severity groups, as suggested by Neblett et al. [18]. In addition, patients were divided into severity subgroups based on Oswestry Disability Questionnaire scores to further evaluate the association between perceived disability and FACS scores.

In addition to the chronic pain clinical sample, FACS-Serb data were collected from a comparison sample of subjects reporting acute pain only (pain for less than three-months). They were recruited from the staff and their families of the Faculty of Medicine, University of Novi Sad, Clinical Centre of Vojvodina. A total of 260 agreed to participate and signed an informed consent. Of the 260-subject sample, 68 reported an acute pain condition and completed the FACS and self-reported pain intensity ratings (see Fig 1). Three pain ratings were collected, including present pain intensity, the strongest pain intensity during the past four-weeks, and the average pain intensity during the past four-weeks. Pain intensity was indicated by each subject with a numerical rating scale (NRS), ranging from 0 (no pain) to 10 (the worst possible pain). Painful body parts included low back, neck, knee, and head.

### Patient-reported clinical variables

In addition to the FACS, four other PRO measures, which assessed FA-related psychosocial variables, were administered: The Pain Catastrophizing Scale (PCS); Beck Depression Inventory (BDI); State-Trait Anxiety Inventory (STAI); and the Oswestry Disability Index (ODI). The PCS measures catastrophic thinking in relation to pain experiences [16]. It contains 13 items, which are rated on a 5-point Likert scale, from 0 (Not at all) to 4 (All the time), with a total score range of 0 to 52. The PCS was also divided into subscale scores, including “rumination” (sum of items 8, 9, 10, 11), “magnification” (sum of items 6, 7, 13), and “helplessness” (sum of items 1, 2, 3, 4, 5, 12). Depressive symptoms were assessed with the BDI, scored from 0 to 63, where higher scores indicate a higher degree of depressive characteristics [24]. Self-reported presence and severity of symptoms of anxiety were assessed with the STAI. There are two subscales within this Inventory: the State Anxiety Scale (STAI-S), which evaluates the current state of anxiety; and the Trait Anxiety Scale (STAI-T), which evaluates the relatively stable feature of anxiety. The STAI has 40 items, 20 items for each subscale scored from 20 to 80, where higher scores indicate greater anxiety [25, 26]. The ODI has 10 items, scored from 0 to 100, which measures perceived disability from activities of daily living [27–29]. Furthermore, patients were divided into five ODI severity subgroups (0–20% “minimal disability;” 21–40% “moderate disability;” 41–60% “severe disability;” 61–80 “crippled;” and 81–100% “bed-bound patients”) as suggested by Fairbanks et al. [29]. As with the acute pain comparison subjects, three measures of patient-reported pain intensity were collected from the chronic pain clinical subjects, including present pain intensity, the strongest pain intensity during the past four-weeks, and the average pain intensity during the past four-weeks. Pain intensity was indicated by each subject with the numerical rating scale (NRS), from 0 (no pain) to 10 (the worst possible pain).

### Statistical analyses

Means and standard deviations of the demographic variables were determined with descriptive statistical analyses. The normality and distribution of the sample were also evaluated for skewness, kurtosis and histograms.

The original 2-factor model suggested by Neblett et al. [19] was tested with confirmatory factor analysis (CFA) for the ordinal data. In this original 2-factor model, Factor 1 represented “general fear avoidance,” and included Items 1–14. Factor 2 represented “types of activities that are avoided,” and included Items 15–20. The model-fit indices included chi-square (χ^2^), comparative fit index (CFI), and root mean square error of approximation (RMSEA). For RMSEA, values of 0.08 or below indicate a close fit [30], and values in the range from 0.08 to 0.10 indicate an acceptable fit [31]. In addition, Cronbach's α coefficients were determined at an anticipated value range of 0.80–0.95 [32–34]. Reliability was calculated with test-retest Intraclass Correlation Coefficients Type 2,1 (ICC_2.1_). The criteria used for Pearson’s r correlation coefficients were: small (0.1≤r≤0.29); medium (0.3 ≤ r ≤ 0.49); and large (0.5 ≤ r ≤ 1) [35].

An error range of 0±10% was used in determining the test-retest reliability. The standard error of measurement (SEM) was determined with the formula: SEM = s√(1–r), where s = mean and standard deviation (SD) of Time 1 and Time 2; r = reliability coefficient for the test and Pearson’s correlation coefficient between test and retest values. A minimal detectable change (MDC_90_) analysis, previously described by Stratford [36], was then calculated using the formula: MDC_90_ = SEM×√2×1.96. Differences among the patient subgroups were tested with either ANOVAs or Kruskal-Wallis tests, as appropriate to the distribution. When assumptions of homogeneity of variance were confirmed, an ANOVA was used with post hoc Tukey. Otherwise, a non-parametric approach was used, with a Kruskal-Wallis test, and a post-hoc Mann Whitney test, with Bonferroni correction of p values. A chi square test was used to determine whether the distributions of categorical variables differed from one another. A priori analysis, conducted with G*Power 3.1, specified a minimum of 321 patients to detect a 0.2 effect size, with α = 0.05 and a power of 0.8 [37, 38]. Data analyses were performed using the IBM SPSS Statistics for Macintosh (version 22.0. Armonk, NY: IBM Corp.) and LISREL 8.80.

## Results

### Score distribution

The mean FACS-Serb score for the total 322 patient sample was 54.93, with a standard deviation of 22.71 and a range of 0 to 97. Scores were normally distributed. The number and percentage of subjects in each severity subgroup were: Subclinical (n = 25, 7.8%); Mild (n = 65, 20.2%); Moderate (n = 84, 26.1%); Severe (n = 104, 32.3%); and Extreme (n = 44, 13.7%).

### Test-retest reliability

The subgroup of 118 patients performed a retest after 7±1 days. These patients did were not statistically different from the rest of the tested subjects in age (51.90±12.16 vs 53.61±12.42, t = 1.203, p = 0.230), female gender (67.8% vs 66.7%, χ^2^ = 0.043, p = 0.835) or FACS score (54.67±23.58 vs 55.08±22.25, t = 0.155, p = 0.877). ICCs were high for the FACS-Serb total score (ICC_2,1_ = 0.928), as well as for Factor 1 (ICC_2,1_ = 0.922) and Factor 2 (ICC_2,1_ = 0.847). Table 1 provides more detailed results.

**Table 1:** Intraclass correlation coefficients and 95% confidence interval (Lower-Upper Bound) of Test-retest reliability.

Item	Intraclass Correlation	95% Confidence Interval (Lower-Upper Bound)
1	0.666	0.519–0.768
2	0.665	0.507–0.771
3	0.776	0.677–0.844
4	0.809	0.723–0.868
5	0.682	0.541–0.779
6	0.882	0.830–0.918
7	0.801	0.714–0.862
8	0.847	0.779–0.894
9	0.829	0.754–0.881
10	0.842	0.770–0.891
11	0.647	0.492–0.755
12	0.693	0.559–0.787
13	0.785	0.691–0.851
14	0.886	0.836–0.921
15	0.707	0.578–0.796
16	0.805	0.719–0.864
17	0.807	0.722–0.866
18	0.800	0.712–0.861
19	0.723	0.606–0.810
20	0.688	0.551–0.783
F1^a^	0.922	0.886–0.946
F2^b^	0.847	0.779–0.893
Total FACS-Serb Score	0.928	0.896–0.950

a”General Fear Avoidance”

^b^ “Types of Activities that are Avoided”

### Factor analysis and internal consistency

**Fig 2:**
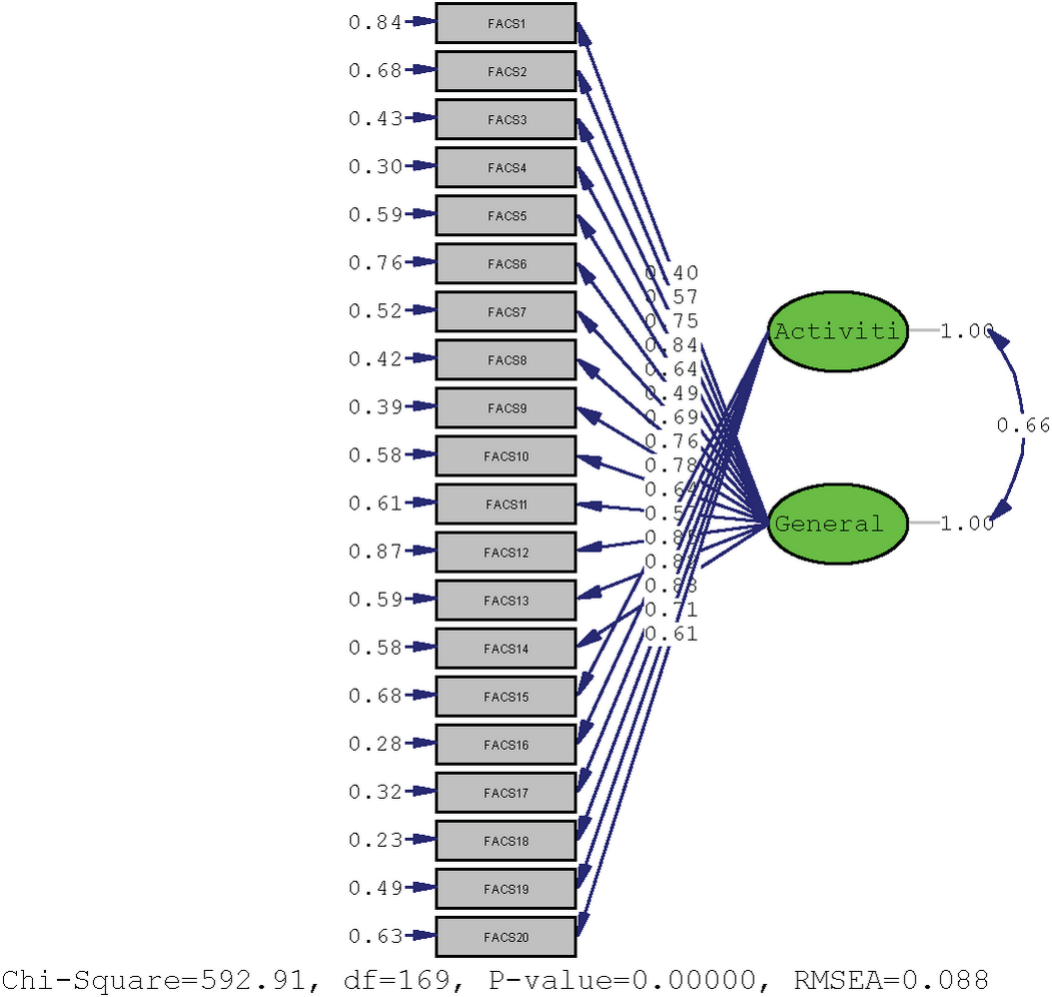
Factor loadings and goodness of fit indexes for underlying two factors of the FACS-Serb.

The original 2-factor model suggested by Neblett et al. [19] was tested with a confirmatory factor analysis (CFA) with ordinal data. The fit indices indicated an acceptable fit: χ^2^ = 592.91, p<0.001, RMSEA = 0.088 and CFI = 0.96 (Fig 2). Internal consistency was excellent for Factor 1 (Cronbach α = 0.904), and acceptable for the Factor 2 (Cronbach α = 0.880).

### Differences in patient-reported clinical variables by FACS severity groups

As shown in Table 2, the sample was divided into FACS-Serb severity groups. Group validity was assessed by testing of the FACS-Serb ability to distinguish between groups of people, on demographic and clinical variables, according to severity of the symptoms. The severity groups did not significantly differ in gender (χ^2^ = 9.177, p = 0.057), but they differed in age (Kruskal Wallis χ^2^ = 15.451, p = 0.004). Therefore, an ANCOVA was performed (with age as the covariate), and a Bonferroni-corrected post hoc analysis was conducted. In addition, because the presumption of homogeneity was not fulfilled for present pain intensity, and the strongest pain in the last four-weeks, Kruskal Wallis analyses were conducted for those variables. As detailed in Table 2, all PRO measures, including pain intensity scores and PCS subscale scores, were significantly different among the FACS-Serb severity groups (p<0.001).

**Table 2:** Patient-reported clinical variables by FACS-Serb severity groups.

Patient-Reported Variables mean (SD)	Total (0–100)	FACS-Serb Severity Groups^h^					χ^2^/F	p	Effect size (Partial Eta Squared)^g^
		Subclinical (0–20)^a^	Mild (21–40)^b^	Moderate (41–60)^c^	Severe (61–80)^d^	Extreme (81–100)^e^			
**N (%)**	322 (100)	25 (7.8)	65 (20.2)	84 (26.1)	104 (32.3)	44 (13.7)			
**Present pain intensity**	5.66 (2.37)	3.24 (2.20)^**b**^^,^^**c**^^,^^**d**^^,^^**e**^	5.03 (2.66)^**a**^^,^^**e**^	5.51 (2.00)^**a**^^,^^**e**^	6.10 (2.06)^**a**^^,^^**e**^	7.18 (1.97)^**a**^^,^^**b**^^,^^**c**^^,^^**d**^	χ^2^ = 49.380 ^f^	<0.001	
**The strongest pain intensity during the past 4 weeks**	7.99 (1.82)	6.56 (2.14)^**d**^^,^^**e**^	7.48 (1.94)^**d**^^,^^**e**^	7.88 (1.69)^**e**^	8.29 (1.73)^**a**^^,^^**b**^	9.09 (0.94)^**a**^^,^^**b**^^,^^**c**^	χ^2^ = 38.300 ^f^	<0.001	
**Average pain intensity during the past 4 weeks mean**	6.54 (1.89)	4.80 (1.38)^**c**^^,^^**d**^^,^^**e**^	5.92 (1.95)^**d**^^,^^**e**^	6.39 (1.60)^**a**^^,^^**e**^	6.81 (1.82)^**a**^^,^^**b**^^,^^**e**^	8.07 (1.48)^**a**^^,^^**b**^^,^^**c**^^,^^**d**^	F = 17.062	<0.001	0.178
**PCS total score**	27.02 (14.02)	6.12 (6.91)^**b**^^,^^**c**^^,^^**d**^^,^^**e**^	16.31 (8.63)^**a**^^,^^**c**^^,^^**d**^^,^^**e**^	24.46 (8.94)^**a**^^,^^**b**^^,^^**d**^^,^^**e**^	33.67 (9.42)^**a**^^,^^**b**^^,^^**c**^^,^^**e**^	43.84 (11.21)^**a**^^,^^**b**^^,^^**c**^^,^^**d**^	F = 100.578	p<0.001	0.560
**PCS Rumination subscale**	9.78 (5.19)	2.36 (2.86)^**b**^^,^^**c**^^,^^**d**^^,^^**e**^	6.91 (3.86)^**a**^^,^^**c**^^,^^**d**^^,^^**e**^	9.14 (3.23)^**a**^^,^^**b**^^,^^**d**^^,^^**e**^	11.72 (3.31)^**a**^^,^^**b**^^,^^**c**^^,^^**e**^	14.89 (6.85)^**a**^^,^^**b**^^,^^**c**^^,^^**d**^	F = 50.556	p<0.001	0.390
**PCS Magnification subscale**	5.89 (3.49)	1.60 (2.08)^**c**^^,^^**d**^^,^^**e**^	3.09 (2.30)^**c**^^,^^**d**^^,^^**e**^	5.17 (2.41)^**a**^^,^^**b**^^,^^**d**^^,^^**e**^	7.47 (2.61)^**a**^^,^^**b**^^,^^**c**^^,^^**e**^	10.11 (2.27)^**a**^^,^^**b**^^,^^**c**^^,^^**d**^	F = 86.565	p<0.001	0.523
**PCS Helplessness subscale**	11.34 (6.48)	2.16 (2.73)^**b**^^,^^**c**^^,^^**d**^^,^^**e**^	6.31 (3.78)^**a**^^,^^**c**^^,^^**d**^^,^^**e**^	10.16 (4.59)^**a**^^,^^**b**^^,^^**d**^^,^^**e**^	14.48 (4.87)^**a**^^,^^**b**^^,^^**c**^^,^^**e**^	18.84 (4.41)^**a**^^,^^**b**^^,^^**c**^^,^^**d**^	χ^2^ = 175.593	p<0.001	-
**ODI**	32.70 (16.94)	13.53 (8.48)^**b**^^,^^**c**^^,^^**d**^^,^^**e**^	23.21 (13.26)^**a**^^,^^**d**^^,^^**e**^	30.19 (12.14)^**a**^^,^^**d**^^,^^**e**^	37.18 (15.17)^**a**^^,^^**b**^^,^^**c**^^,^^**e**^	50.83 (16.00)^**a**^^,^^**b**^^,^^**c**^^,^^**d**^	F = 36.345	p<0.001	0.321
**BDI**	12.32 (9.30)	4.20 (3.02)^**c**^^,^^**d**^^,^^**e**^	7.16 (5.43)^**c**^^,^^**d**^^,^^**e**^	11.06 (7.24)^**a**^^,^^**b**^^,^^**d**^^,^^**e**^	14.59 (8.70)^**a**^^,^^**b**^^,^^**c**^^,^^**e**^	21.50 (11.61)^**a**^^,^^**b**^^,^^**c**^^,^^**d**^	χ^2^ = 92.573 ^f^	P<0.001	
**STAI S**	40.28 (11.78)	30.52 (6.57)^**c**^^,^^**d**^^,^^**e**^	34.06 (8.83)^**d**^^,^^**e**^	38.87 (11.35)^**a**^^,^^**d**^^,^^**e**^	43.77 (10.87)^**a**^^,^^**b**^^,^^**c**^	49.32 (11.51)^**a**^^,^^**b**^^,^^**c**^	χ^2^ = 75.049 ^f^	P<0.001	
**STAI T**	41.46 (11.24)	32.80 (6.78)^**c**^^,^^**d**^^,^^**e**^	34.78 (8.63)^**c**^^,^^**d**^^,^^**e**^	39.96 (9.52)^**a**^^,^^**b**^^,^^**d**^^,^^**e**^	44.75 (10.57) ^**a**^^,^^**b**^^,^^**c**^^,^^**e**^	51.16 (11.14)^**a**^^,^^**b**^^,^^**c**^^,^^**d**^^,^	F = 24.103	P<0.001	0.235

^a,b,c,d,e^ Groups (different symbols) that significantly differed from each other in continuous comparisons.

^f^The Kruskal-Wallis test was applied, and the χ2 value is reported because the assumption of homogeneity of variances was not met for this variable

^g^Effect size (partial eta squared): 0.01 = small; 0.06 = medium; 0.14 = large

^h^FACS-Serb, Serbian version of Fear-Avoidance Component Scale.

### Differences in patient-reported clinical variables by ODI severity groups

From the total number of subjects, nine did not complete the ODI so they were excluded for further analysis. Subgrouping of subjects by ODI scores revealed: 81 (25.9%) subjects within the”minimal disability”range; 138 (44.1%) within the”moderate disability”range; 77 (23.9%) within the “severe disability” range; 14 (4.5%) within the “crippled” range; and 3 (1%) subjects within the “bed-bound” range. Due to small number of subjects in the last 3 groups, they were merged into a single “severe disability”group (n = 94; 30%), leaving 3 ODI severity groups for analyses (i.e., mild, moderate, and severe). Gender, age, and the FACS-Serb scores were compared among these three ODI severity groups. These groups did not significantly differ in gender (χ^2^ = 1.044, p = 0.593) but they differed in age (χ^2^ = 16.143, p<0.001). Therefore, an ANCOVA was performed, and a Bonferroni-corrected post hoc analysis was conducted. For the total FACS-Serb score, the assumption of homogeneity was not fulfilled, so a Kruskal-Wallis test was used, and a Mann-Whitney test for post hoc analysis was performed. The FACS-Serb total score, as well as the individual Factor scores, significantly differed among the three ODI severity subgroups (χ^2^ = 99.215, p<0.001; F = 49.210, p<0.001; F = 51.075, p<0.001, respectively), as detailed in Table 3.

**Table 3:** FACS-Serb total scores and subscale factor scores by ODI severity groups.

Patient-Reported Variables mean (SD)	Total	ODI (0–20%)^a^ Minimal disability	ODI (21–40%)^b^ Moderate disability	ODI (41–100%)^c^ Severe disability	F/χ^2^	p	Effect size (Partial Eta Squared)^e^
**N (%)**	313 100)	81 (25.9)	138 (44.1)	94 (30.0)	-	-	-
**FACS**^**f**^ **total score**	55.28 (22.53)	37.20 (20.22)^**b**^^,^^**c**^	54.99 (18.97)^**a**^^,^^**c**^	71.30 (16.64)^**a**^^,^^**b**^	χ^2^ = 99.215 ^d^	<0.001	-
**FACS Factor 1**	40.22 (15.94)	28.54 (14.40)^**b**^^,^^**c**^	40.13 (13.86) ^**a**^^,^^**c**^	50.40 (12.95) ^**a**^^,^^**b**^	F = 49.210	<0.001	0.242
**FACS Factor 2**	15.06 (8.79)	8.65 (8.13)^**b**^^,^^**c**^	14.86 (7.43) ^**a**^^,^^**c**^	20.89 (7.13) ^**a**^^,^^**b**^	F = 51.075	<0.001	0.248

^a,b,c^ Groups (different symbols) that significantly differed from each other in continuous comparisons.

^d^The Kruskal-Wallis test was applied, and the χ2 value is reported because the assumption of homogeneity of variances was not met for these variables.

^e^Effect size (partial eta squared): 0.01 = small; 0.06 = medium; 0.14 = large

^f^FACS-Serb, Serbian version of Fear-Avoidance Component Scale.

### Differences in FACS scores between the chronic pain clinical sample and the acute pain comparison sample

As shown in Table 4, the FACS-Serb scores were compared between the chronic pain clinical group and the acute pain comparison group. These groups did not significantly differ in gender (χ^2^ = 0.819, p = 0.404), but they differed in age (t = 2.354, p = 0.021). Therefore, an ANCOVA (with age as a covariate) was performed, and a Bonferroni-corrected post hoc analysis was conducted. The Factor-2 assumption of homogeneity was not fulfilled (Levene’s test indicated unequal variances: F = 7.908, p = 0.005), so a Mann-Whitney test was performed. The FACS-Serb total score, as well as the individual Factor scores, differed significantly between the chronic and acute pain groups (F = 70.443, p<0.001; F = 70.077, p<0.001; χ^2^ = 5490.500, p<0.001, respectively).

**Table 4:** FACS-Serb score comparison between the chronic pain clinical sample (N = 322) and the acute pain control sample (N = 68).

Variables mean (SD)	Chronic Pain Clinical Group	Acute Pain Comparison Group	t/F/χ^2^	p	Effect size (Partial Eta Squared)^b^
**Female gender n (%)**	216 (67.1)	41 (60.3)	χ^2^ = 1.151	0.4325	-
**Age in years**	52.99 (12.33)	48.44 (16.78)	t = 2.116	0.037	-
**Present pain intensity**	5.66 (2.37)	2.49 (1.74)	χ^2^ = 3194.500 ^a^	<0.001	-
**The strongest pain intensity during the past 4 weeks**	7.99 (1.82)	3.96 (2.06)	F = 251.395	<0.001	0.394
**Average pain intensity during the past 4 weeks**	6.54 (1.89)	3.03 (1.68)	F = 190.961	<0.001	0.330
**FACS-Serb** ^**c**^ **total score**	54.93 (22.71)	28.75 (20.75)	F = 69.406	<0.001	0.152
**FACS-Serb Factor 1**	40.05 (16.08)	21.38 (14.97)	F = 70.415	<0.001	0.154
**FACS-Serb Factor 2**	14.88 (8.82)	7.13 (6.94)	χ^2^ = 5401.500 ^a^	<0.001	

^a^The Mann-Whitney test was applied, and the χ2 value is reported here because the assumption of homogeneity of variances was not met for this factor

^b^Effect size (partial eta squared): 0.01 = small; 0.06 = medium; 0.14 = large

^c^FACS-Serb, Serbian version of Fear-Avoidance Component Scale

### Intercorrelations among all patient-reported clinical variables

Table 5 presents correlations among all of the clinical variables for the chronic pain clinical sample (e.g. convergent validity), including scores on pain intensities, PCS, ODI, BDI, STAI-S, STAI-T, total FACS-Serb, and both Factor1 and Factor 2. All correlations were significant (p<0.01).

**Table 5:** Correlations among patient-reported variables.

	Pain Current	Pain Max	Pain Average	PCS	ODI	FACS Total	FACS Factor 1	FACS Factor 2	BDI	STAI S	STAIT
**Pain Current**	-										
**Pain Max**	0.629	-									
**Pain Average**	0.691	0.721	-								
**PCS**	0.420	0.344	0.456	-							
**ODI**	0.523	0.373	0.441	0.555	-						
**FACS Total**	0.408	0.367	0.429	0.772	0.592	-					
**FACS Factor 1**	0.386	0.349	0.424	0.778	0.543	0.954	-				
**FACS Factor 2**	0.347	0.310	0.332	0.570	0.532	0.837	0.633	-			
**BDI**	0.299	0.190	0.275	0.556	0.591	0.534	0.545	0.381	-		
**STAI S**	0.273	0.146	0.237	0.488	0.491	0.480	0.502	0.322	0.720	-	
**STAI T**	0.244	0.149	0.231	0.546	0.473	0.505	0.543	0.310	0.788	0.835	-

All Correlations were significant at the 0.01 level (2-tailed)

## Discussion

Several well-recognized FA-related PRO measures are currently available [6, 7, 15, 16], but they have been criticized for psychometric weaknesses, lack of evidence-based cut-off scores, poor item specificity, and not addressing all of the important components of the current FA model [17, 39, 40]. With the increased interest in FA among pain specialists, and the continued evolution of the FA model, the need for an up-to-date evaluation tool for this construct increases. The FACS was designed to encompass important cognitive, emotional, and behavioral components of the current FA model, including components from previous FA-related PRO measures, into a brief but comprehensive instrument for persons with painful medical conditions [18, 19]. In the current study, the original English version was translated into Serbian, in accordance with IPSOR Guidelines [23], then was psychometrically-validated in a group of chronic pain subjects and a separate group of acute pain comparison subjects. Overall, it was found that the FACS-Serb scores were distributed normally in the 322-patient sample, with a mean score of 54.93, standard deviation of 22.71, and range of 0 to 97. The developers of the original FACS suggested a 2-factor solution that explained 52% of the total variance [19]. The first Factor represented “general FA,” and encompassed items 1–14, while the second Factor represented “activities that are avoided,” and encompassed items 15–20. The CFA of the FACS-Serb in the present study revealed an acceptable fit of this original 2-factor model. Internal consistency was also excellent for Factor 1, but merely acceptable for Factor 2. However, the somewhat lower value of Cronbach α for the Factor 2 was not a surprise due to the relatively small number of items in this Factor [41]. Test-retest reliability showed excellent results for FACS-Serb total score and Factor 1 and Factor 2, respectively, which was similar to earlier findings [19]. All of the above reviewed results confirmed a psychometrically-sound basis of the FACS-Serb.

Previous studies have found associations among other measures of FA and: patient-reported pain intensity [42–44]; catastrophizing [45, 46], depressive symptoms [47–49], anxiety [2, 50], and perceived disability [51, 52]. To further evaluate the construct validity of the FACS-Serb, all of these variables were again assessed in the present study. Subjects completed the BDI, PCS, STAI, and ODI, and a numeric rating scale of current, strongest, and average pain over the last four-weeks. PCS subscale scores (Rumination, Magnification, and Helplessness) were also collected. To evaluate these variables, patients were first divided into one of five-FACS severity groups (from Subclinical to Extreme), based on total FACS-Serb scores, as recommended by Neblett et al. [18]. Significant differences were found among all of the variables, so that lower FACS-Serb severity was associated with lower scores, and higher FACS-Serb severity was associated with higher scores, on all of the other PRO measures. In addition, most of the scores on these variables were significantly different among the individual FACS-Serb severity subgroups, in a “stair-step pattern,” from lower to higher scores, as the FACS-Serb severity increased. Neblett et al. [18, 19] have previously identified similar associations among FACS scores and FA-related PRO measures of pain intensity, depressive symptoms, perceived disability, perceived injustice, insomnia, and kinesiophobia (i.e., TSK scores) [18, 19].

It has been suggested that earlier versions of the FA model underplayed the role of pain intensity [53]. However, newer studies have found a relationship between FA and intensity of the pain [42–44]. Our results confirmed these findings. Positive correlations among FACS-Serb scores and pain intensity measures were significant, although not as strong as with the other FA-related variables that we investigated. Similar results were found by Kroska, using other measures of FA, in his meta-analysis [5]. Interestingly, the original psychometric evaluation of the English FACS, in a group of chronic musculoskeletal pain disorder patients, found that pain intensity was not significantly different among FACS severity groups after correcting for Type I error. Perhaps, the intensity of pain is somewhat less related to FA than perceived pain-related disability. Indeed, there is evidence that pain-related fear is more disabling than pain severity [53]. Previous studies have found that disability is more often the reason for doctor-visits than pain intensity [54, 55], suggesting that it may not be pain itself, but its interference with daily activities, that motivates patients to seek healthcare [4, 56].

There is a strong relationship between FA beliefs and disability within the FA model [4]. This connection has been confirmed by many authors [51, 52, 57–59]. For instance, patients reporting higher pain-related fear have been found to perform more poorly on physical tasks in comparison to patients reporting lower pain-related fear [3, 53]. A relationship between performance and FACS scores has also been demonstrated. FACS scores were highly and inversely correlated with objective lifting performance in a cohort of chronic musculoskeletal pain patients in a functional restoration treatment program [19]. To further investigate the association between FACS-Serb scores and perceived disability, the chronic pain sample in the present study was divided into three disability severity groups, based on ODI scores. Fairbanks et al. [29] suggested subgrouping of patients into five subgroups according to the ODI scores, graded from minimal disability to bed-bound patients. However, because the total number of patients in the third, fourth, and fifth groups was too small in the present study, they were merged into a single “severe disability” subgroup, creating three ODI severity groups. Large, positive, and significant correlations between ODI and FACS-Serb scores were observed. Total FACS-Serb and individual Factor scores were also significantly different among all three disability severity groups. FACS-Serb scores again increased in a “stair-step” fashion, from lower to higher perceived disability.

As stated earlier, the FA model asserts that FA beliefs in response to acute pain can lead to a transition into chronic pain and disability. [1, 2, 60]. It can therefore be assumed that chronic pain subjects should report a higher level of FA than acute pain subjects. To investigate the discriminate validity of the FACS-Serb, the total and Factor scores, as well as pain intensity ratings, were compared between the chronic pain cohort and a separate cohort of acute pain subjects. Our results showed that the acute pain group scored significantly lower on the FACS-Serb and pain measures than the chronic pain group.

It should be noted that, in any large study of this type, there are usually some limitations. In the present investigation, the chronic pain patient sample was recruited from a single hospital system in Serbia. Therefore, these results may not generalize to other patient populations. Also, to the best of our knowledge, the only FA-specific PRO measure that was available in Serbian was the PCS, so we were unable to investigate associations with other FA-specific measures, such as the TSK. In addition, due to sample-size limitations, we relied on exploratory factor analysis from earlier studies, and we were not able to perform our own exploratory factor analysis. Sample size has also been a limitation in earlier FACS exploratory factor analysis [19]. In order to overcome this limitation, some authors of this paper have begun a project of collecting a pool of data from a larger sample of subjects from multiple countries and languages. This project is in progress, and it is hoped that it will show a definitive factor structure of the FACS (personal communications).

In conclusion, this was the first translation, cross-cultural adaptation, and validation study of the FACS into the Serbian language. The FACS-Serb showed satisfactory internal consistency and reliability, which corresponded with previous analyses of the FACS [18, 19]. Confirmatory factor analyses also corresponded well to the original English version. Convergent validity of the FACS-Serb was demonstrated by positive correlations with PCS, BDI, STAI, ODI and pain intensity scores. Discriminant validity of the FACS-Serb was demonstrated through its ability to distinguish between perceived disability subgroups (using ODI scores), and between acute and chronic pain subjects. It is anticipated that the FACS-Serb will be a useful pain assessment tool for Serbian-speaking health professionals.

## Supporting information

S1 Appendix Serbian version of Fear-Avoidance Component Scale (FACS-Serb). (PDF)
