# Supplementary material for: Psychometric validation of the Serbian version of the Fear Avoidance Component Scale (FACS)
Source: PLoS One. 2018 Sep 24;13(9):e0204311. doi: 10.1371/journal.pone.0204311 (PMC6152979; doi:10.1371/journal.pone.0204311)
Supplement: S1 Appendix — (PDF) [file pone.0204311.s001.pdf]

Skala komponenti izbegavanja aktivnosti usled straha (FACS-Serb)

Ime: ______________________________________ ID: ______________ Datum:_________

Uputstva: Ljudi različito reaguju na bol. Želeli bismo da saznamo šta Vi mislite o tome i kako se osećate u vezi sa svojim bolnim stanjima, kao i to na koji način bol utiče na intenzitet vaših aktivnosti. Molimo Vas da razmislite o tome kako ste se osećali tokom protekle nedelje, i da zaokružite jedan broj od 0 do 5 na skali ispod kao odgovor na svako od pitanja.

5= U potpunosti se slažem
4= Uglavnom se slažem
3= Delimično se slažem
2= Delimično se ne slažem
1= Uglavnom se ne slažem
0= U potpunosti se ne slažem

**Tokom protekle nedelje, do koje mere biste se složili s izjavama u vezi sa svojim bolnim stanjem?**	*U potpunosti se slažem*	*Uglavnom se slažem*	*Delimično se slažem*	*Delimično se ne slažem*	*Uglavnom se ne slažem*	*U potpunosti se ne slažem*
1) Trudim se da izbegavam pokrete i aktivnosti zbog kojih mi se bol pojačava..	5	4	3	2	1	0
2) Zabrinut sam zbog bolova koje osećam.................	5	4	3	2	1	0
3) Verujem da će mi se bol pojačavati sve dok me potpuno ne onesposobi..	5	4	3	2	1	0
4) Osećam jak strah kad pomislim na svoje bolno stanje...	5	4	3	2	1	0
5) Ne pokušavam da se bavim određenim aktivnostima jer se plašim da ću se povrediti (ponovo povrediti)...	5	4	3	2	1	0
6) Kad imam zaista jake bolove, takođe imam i simptome poput mučnine, poteškoća s disanjem, lupanja srca, drhtavice i/ili vrtoglavice................	5	4	3	2	1	0
7) Nepravedno je što moram da živim s bolom........	5	4	3	2	1	0
8) Zbog svog bolnog stanja do kraja života izložen sam riziku od budućih povreda (ili ponovnih povreda)..	5	4	3	2	1	0

	Tokom protekle nedelje, do koje mere biste se složili s izjavama u vezi sa svojim bolnim stanjem?	U potpunosti se slažem	Uglavnom se slažem	Delimično se slažem	Delimično se ne slažem	Uglavnom se ne slažem	U potpunosti se ne slažem
9)	Zbog bolova koje osećam moj život će se promeniti zauvek..	5	4	3	2	1	0
10)	Nemam kontrolu nad svojim bolom........................	5	4	3	2	1	0
11)	Ne pokušavam da vršim određene aktivnosti i pokrete jer se plašim da će mi se bol pogoršati....	5	4	3	2	1	0
12)	Neko drugi je kriv za moje bolno stanje...................	5	4	3	2	1	0
13)	Bol usled mog zdravstvenog stanja je znak upozorenja da sa mnom nešto ozbiljno nije u redu	5	4	3	2	1	0
14)	Niko ne shvata koliko su jaki bolovi koje imam.........	5	4	3	2	1	0

	Započnite svaki od sledećih iskaza ovom izjavom: Tokom protekle nedelje, zbog svog bolnog stanja, izbegavao sam sledeće:	U potpunosti se slažem	Uglavnom se slažem	Delimično se slažem	Delimično se ne slažem	Uglavnom se ne slažem	U potpunosti se ne slažem
15)	…naporne aktivnosti (poput teškog rada u bašti ili pomeranja teškog nameštaja)................................	5	4	3	2	1	0
16)	...umerene aktivnosti (poput spremanja večere ili čišćenja kuće)...	5	4	3	2	1	0
17)	...lagane aktivnosti (poput odlaska u bioskop ili na ručak)..	5	4	3	2	1	0
18)	...sve svoje obaveze kod kuće i/ili na poslu............	5	4	3	2	1	0
19)	...rekreaciju i/ili vežbanje (stvari koje radim iz zabave i radi zdravlja)...	5	4	3	2	1	0
20)	...aktivnosti prilikom kojih moram da koristim delove tela koji su bolni..	5	4	3	2	1	0

Ukupno:
